# Integrative transcriptome and proteome analyses provide new insights into different stages of *Akebia trifoliata* fruit cracking during ripening

**DOI:** 10.1186/s13068-020-01789-7

**Published:** 2020-08-20

**Authors:** Juan Niu, Yaliang Shi, Kunyong Huang, Yicheng Zhong, Jing Chen, Zhimin Sun, Mingbao Luan, Jianhua Chen

**Affiliations:** Institute of Bast Fiber Crops, Chinese Academy of Agricultural Sciences/Key Laboratory of Stem-Fiber Biomass and Engineering Microbiology, Ministry of Agriculture, Xianjiahu West Road, Changsha, 410205 Hunan Province People’s Republic of China

**Keywords:** *Akebia trifoliata*, Pericarp structure, Fruit cracking, Transcriptome, Proteome, Pentose and glucuronate interconversions, Galactose metabolism

## Abstract

**Background:**

*Akebia trifoliata* (Thunb.) Koidz may have applications as a new potential source of biofuels owing to its high seed count, seed oil content, and in-field yields. However, the pericarp of *A. trifoliata* cracks longitudinally during fruit ripening, which increases the incidence of pests and diseases and can lead to fruit decay and deterioration, resulting in significant losses in yield. Few studies have evaluated the mechanisms underlying *A. trifoliata* fruit cracking.

**Results:**

In this study, by observing the cell wall structure of the pericarp, we found that the cell wall became thinner and looser and showed substantial breakdown in the pericarp of cracking fruit compared with that in non-cracking fruit. Moreover, integrative analyses of transcriptome and proteome profiles at different stages of fruit ripening demonstrated changes in the expression of various genes and proteins after cracking. Furthermore, the mRNA levels of 20 differentially expressed genes were analyzed, and parallel reaction monitoring analysis of 20 differentially expressed proteins involved in cell wall metabolism was conducted. Among the molecular targets, pectate lyases and pectinesterase, which are involved in pentose and glucuronate interconversion, and β-galactosidase 2, which is involved in galactose metabolism, were significantly upregulated in cracking fruits than in non-cracking fruits. This suggested that they might play crucial roles in *A. trifoliata* fruit cracking.

**Conclusions:**

Our findings provided new insights into potential genes influencing the fruit cracking trait in *A. trifoliata* and established a basis for further research on the breeding of cracking-resistant varieties to increase seed yields for biorefineries.

## Background

*Akebia trifoliata* (Thunb.) Koidz is a perennial, wild, woody vine, belonging to the family Lardizabalaceae and the subgenus *Akebia Decne* [1]. This species is characterized by numerous seeds (300–800), high seed yield (400 kg/667 m^2^), and high seed oil contents (47.62%) [2–4]. The physicochemical properties of seeds, such as water content, iodine value, acid value, peroxide value, cetane number, flash point, and cold filter plugging point of the oil, meet the biodiesel standard, implying applications in biodiesel production for the development and utilization of liana oil crops [3, 5]. Similar in structure to petrol-diesel, biodiesel prepared with C15–C18 alkane fractions with a high cetane number of 91 can be obtained from *A. trifoliata* seed oil. This cetane number is similar to that of biodiesel prepared using *Sapium sebiferum* oil (40.2) and *Vernicia fordii* (53) [6–8]. Moreover, the fruits of *A. trifoliata* have high sugar content, which is important in industrial bioethanol production because it can boost the amount of ethanol produced at the end of fermentation [9, 10]. Enzymatic saccharification and ethanol yields of 98.0% and 100%, respectively, were achieved from the solid residues of *Akebia* after CHCl-formic acid pretreatment, suggesting potential applications as a feedstock for cellulosic ethanol production [11]. The advantages of *A. trifoliata*, including high seed count, high seed oil contents, abundant yields from the field, high total sugar contents of fruit, wide adaptability, tolerance to both drought and heat, and ease of management, could provide new resources for biodiesels and feedstocks for cellulosic ethanol production [4, 5, 11]. *A. trifoliata* can also be exploited as a new high-value fruit crop owing to its high nutritional, ornamental, economic, medicinal, and potential development value [12, 13].

However, the pericarp of *A. trifoliata* cracks longitudinally along the ventral suture when matured. Fruit cracking is a serious problem that increases the incidence of pests and diseases, leading to fruit decay and deterioration, affecting the utilization rate of fruit and seeds and causing significant losses in yields and commercial value [14, 15]. Studies have indicated that the dry mass and oil contents of insect-infested fruits (33.1 mg/fruit and 3.9%, respectively) are lower than those of healthy fruits (67.4 mg/fruit and 39.9%, respectively); moreover, the observed fruit and oil yields (2.9 and 0.6 kg/tree, respectively) are lower than the excepted yields (4.7 and 1.9 kg/tree, respectively) [16]. Fruit ripening and cracking is a complex, genetically programmed process that is accompanied by the synthesis of large amounts of proteins and the transcription of many genes [17, 18]. Studies have indicated that different genetic accessions exhibit major differences in cracking resistance, suggesting that genetic factors play important roles in sweet cherry fruit cracking resistance [19]. Studies on fruit cracking have been performed in many species, including tomatoes, litchis, durians, and apples; these studies have suggested that cell wall-modifying proteins, such as polygalacturonases (PGs), pectinesterase (PE), β-galactosidases (β-GALs), expansins (EXPs), and xyloglucan endotransglycosylase proteins [14, 20–22], are associated with fruit cracking. The basic helix-loop-helix (bHLH) gene *INDEHISCENT* regulates *Lepidium campestre* fruit dehiscence [23]. Additionally, Dong et al. [24] found that pod shattering resistance in soybeans was mediated by the *NAC* gene. Sorefan et al. [25] indicated that a regulated auxin minimum was required for seed dispersal in *Arabidopsis*. Consequently, a better understanding of the genetic analysis of fruit cracking is necessary to prevent cracking phenotypes.

Although many studies have evaluated fruit cracking, little progress has been made with regard to our understanding of the molecular mechanisms underlying *A. trifoliata* fruit cracking, and only two studies have performed transcriptome sequencing in this species [1, 26]. Our limited knowledge of the molecular characteristics of *A. trifoliata* has made it difficult to recommend preventive measures for fruit cracking. Next-generation sequencing methods, such as transcriptome and proteome technologies for measuring gene expression and protein abundance, have become powerful tools for the discovery of novel genes and their functions in regulating fruit ripening and cracking [27, 28].

Therefore, in this study, an integrative analysis of the transcriptome and proteome was performed to enhance our understanding of *A. trifoliata* fruit cracking during ripening at the molecular level. Our data provide important insights into omics resources and candidate genes responsible for fruit cracking traits in *A. trifoliata*.

## Results

### Changes in pericarp structure

**Fig. 1:**
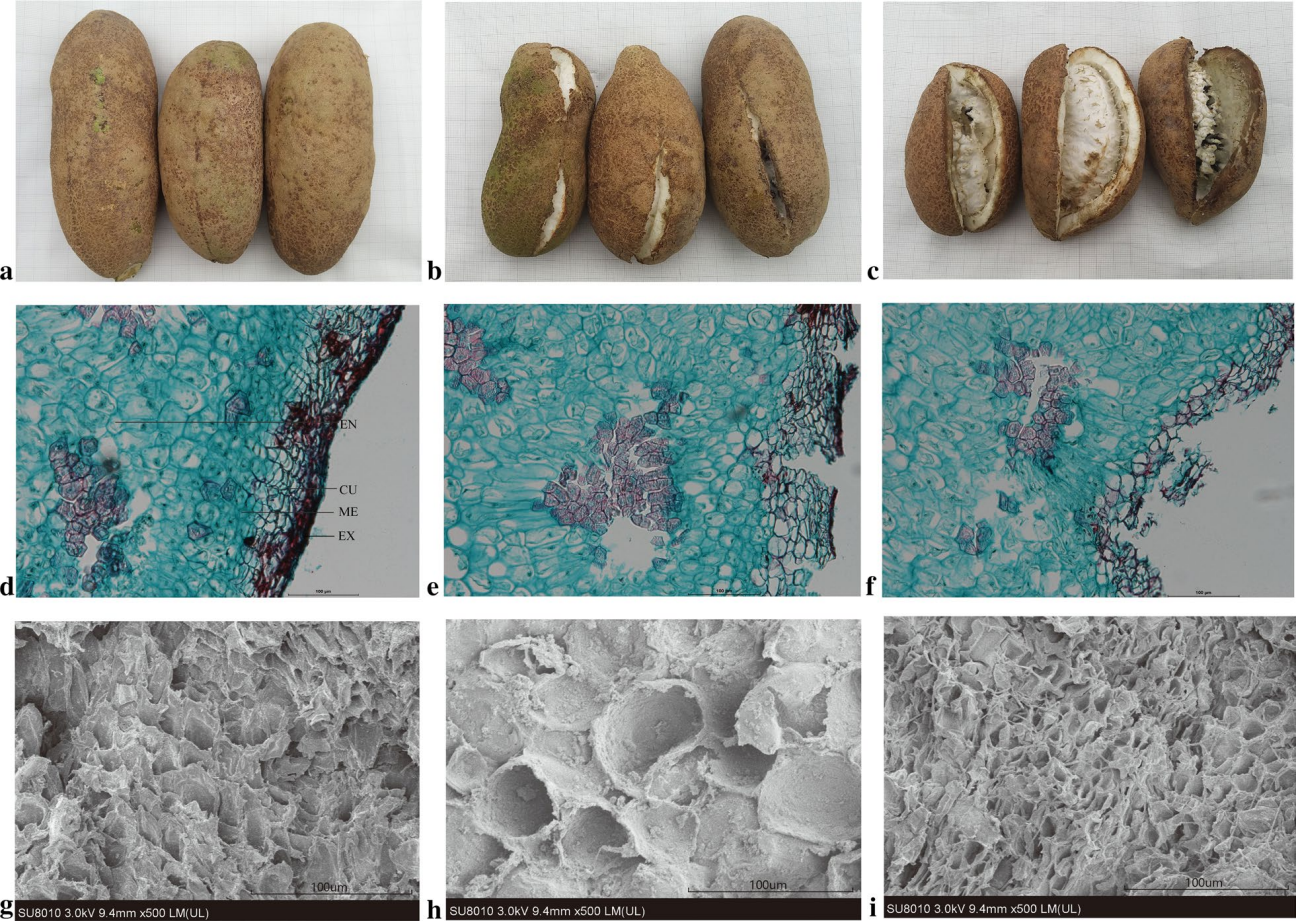
The morphology and structure changes of ‘Nong No.8′ pericarp at different development stages. **a**–**c** The morphology changes of *A. trifoliate* pericarp in non-cracking stage (PS) (**a**), initial cracking stage (PM) (**b**) and total cracking stage (PL) (**c**), respectively. **d**–**f** Pericarp structure of ‘Nong No.8′ at different development stages using the semi-thin slices method and stained with Safranin O-staining in PS (**d**), PM (**e**) and PL (**f**). **g**–**i** Pericarp structure of ‘Nong No.8′ at different development stages using the scanning electron microscope method in PS (**g**), PM (**h**) and PL (**i**). EX. Exocarp; ME. Mesocarp; EN. Endocarp; CU. Cuticle

First, we evaluated the dynamic structures of fruit pericarps during different stages of ripening (Fig. 1). In the non-cracking stage (PS), the pericarp cells and cuticles were densely arranged, with small intercellular spaces and continuous distribution (Fig. 1a, d, g). However, in the initial cracking stage, the cell wall thinned, the cell volume increased, the number of cell layers decreased, and the cells were loosely arranged with poor integrity; moreover, the spacing between the cells increased, and the cell of the exocarp and mesocarp began to degrade in the initial cracking stage (PM; Fig. 1b, e, h). Irregularly arranged layers with continuously reduced numbers and larger spaces were observed as an evidence of cell degradation throughout the cracking stages (PL; Fig. 1c, f, i).

### Transcriptomic analysis overview

Nine cDNA libraries were constructed, and 47.05, 46.92, and 54.00 million raw sequence reads were generated from the PS, PM, and PL libraries, respectively. After removing low-quality reads and adaptor sequences, 46.45, 46.35, and 53.46 million clean reads with 91.58–93.75% Q30 bases and 45.94–48.48% GC content, respectively, were obtained (Additional file 1: Table S1). The resulting *A. trifoliata* transcriptome contained 241,376 transcripts, ranging from 201 to 2000 bp, and 186,054 unigenes (> 200 bp; Table 1 and Additional file 1: Fig. S1).

**Table 1:** Summary of the transcriptome and proteome data in *Akebia trifoliata fruits*

RNA-seq data		MS data based on transcriptome	
Total number of transcripts	241,376	Total spectra	812,625
Mean length of transcripts (bp)	515	Identified spectra	68,151
Total number of unigenes	186,054	Identified peptides	12,456
Mean length of unigenes (bp)	447	Unique peptides	10,572
N50 length of transcripts (bp)	713	Identified proteins	2839
N50 length of unigenes (bp)	518		

All unigenes were annotated using Basic Local Alignment Search Tool (BLAST) searches against the following five databases: National Center for Biotechnology Information nonredundant protein sequences database (NR; 100,329; 53.9% and 41.57% of all identified unigenes and transcripts, respectively), SwissProt (56,346; 30.3% and 23.3%), Protein Families database (Pfam; 34,428; 18.5% and 14.3%), Gene Ontology database (GO; 44,558; 23.9% and 18.5%), and Kyoto Encyclopedia of Genes and Genomes pathway database (KEGG; 30,298; 16.3% and 12.6%). This indicated that the NR database provided the largest number of annotations, suggesting that 100,329 unigenes corresponded with sequences from at least one of the public databases, and 7283 unigenes were annotated to all databases. Moreover, 17,601, 19,281, and 13,525 unique unigenes and 45,866, 48,104, and 43,303 absent unigenes were identified in PS, PM, and PL, respectively. Most of these were uncharacterized, unknown, and hypothetical proteins in the annotation results (Additional file 1: Tables S2–S3).

Among these unigenes, 9301 were identified as differently expressed genes (DEGs), including 2703 (779 up- and 1924 downregulated), 4694 (3815 up- and 879 downregulated) and 1904 co-expressed in PM versus PS and PL versus PM groups, respectively (Table 2), which are presented in a volcano plot (Additional file 1: Fig. S2a–b). The summary information of these DEGs is shown in Additional file 1: Table S4.

**Table 2:** Summary of transcripts and proteins detected from RNA and TMT sequence data

	Transcriptome		Proteome	
	PM_PS	PL_PM	PM_PS	PL_PM
Unique proteins/genes detected	100,329	100,329	2839	2839
Significantly DEGs/DAPs	4607	6598	190	50
Up- regulated	779	3815	75	20
Down- regulated	1924	879	98	13
Shared genes/proteins	1904	1904	17	17
Shared genes/proteins (upregulated)	1123	808	9	8
Shared genes/proteins (downregulated)	781	1096	8	9
Co-regulated DEGs-DAPs	14	4	14	4
Co-regulated DEGs-DAPs with the same trends	12	4	12	4

### Functional classification of identified DEGs

**Fig. 2:**
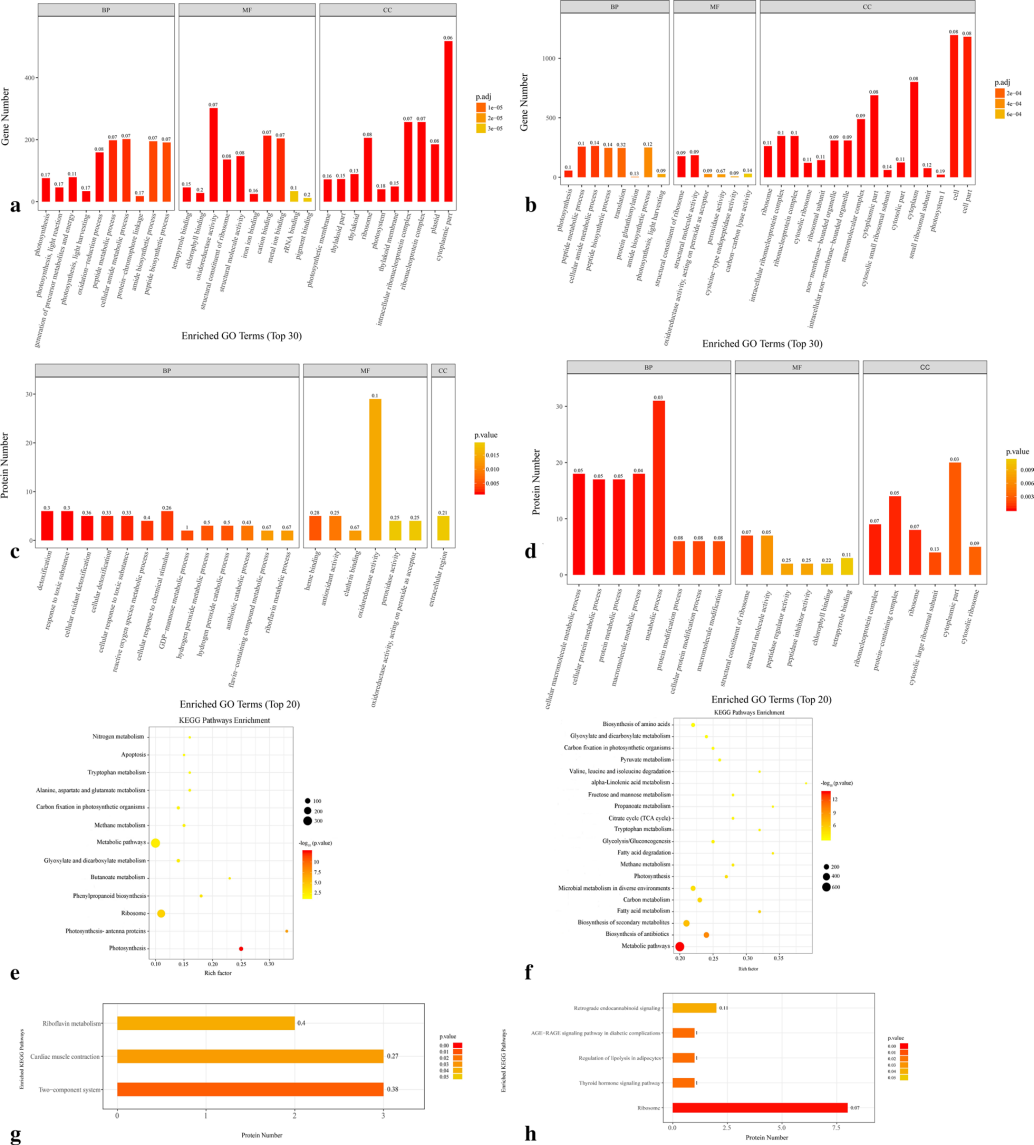
The enrichment analysis of the DEGs and DAPs using GO and KEGG pathways. **a**, **b** GO classifications of DEGs in PM_PS (**a**) and PL_PM (**b**), respectively. The horizontal axis shows the top 30 enriched GO terms in the biological process, molecular function, and cellular component categories. The vertical axis shows the number of genes enriched in each term. **c**, **d** GO classifications of DAPs in PM_PS (**c**) and PL_PM (**d**), respectively. The horizontal axis shows the top 20 enriched GO terms in the biological process, molecular function, and cellular component categories. The vertical axis shows the number of proteins enriched in each term. The height and color of each histogram indicates the number of genes/proteins and *p* values of the enriched term. The label of each histogram indicates the rich factor, which represents the ratio of DEG/DAPs to total genes/proteins identified in GO functional category. **e**, **f** KEGG pathways enrichment of DEGs in PM_PS and PL_PM, respectively. g–h KEGG pathways enrichment of DAPs in PM_PS and PL_PM, respectively. The horizontal axis shows the enrichment factors of the KEGG pathways terms (**e**, **f**), and the number of proteins enriched in each term (**g**, **h**). The vertical axis shows the top enriched KEGG pathways terms. The size and color of the circle indicates the number of genes and *p* values of the enriched term, respectively. The length and color of each histogram indicates the number of proteins and *p* values of the enriched term. The label of each histogram indicates the rich factor, which represents the ratio of DEG/DAPs to total genes/proteins identified in KEGG pathway functional category

Bioinformatics analysis indicated that most DEGs in the GO terms of biological process (BP) were involved in cellular amide metabolic processes and amide metabolic processes. Genes related to structural molecular activity and oxidoreductase activity accounted for the highest proportions of DEGs in the molecular function (MF) category in both the PM_PS and PL_PM groups. Cytoplasmic parts and intracellular ribonucleoprotein complexes comprised the highest proportions of DEGs in the cell components (CCs) in the PM_PS and PL_PM groups, respectively (Fig. 2a, b).

**Fig. 3:**
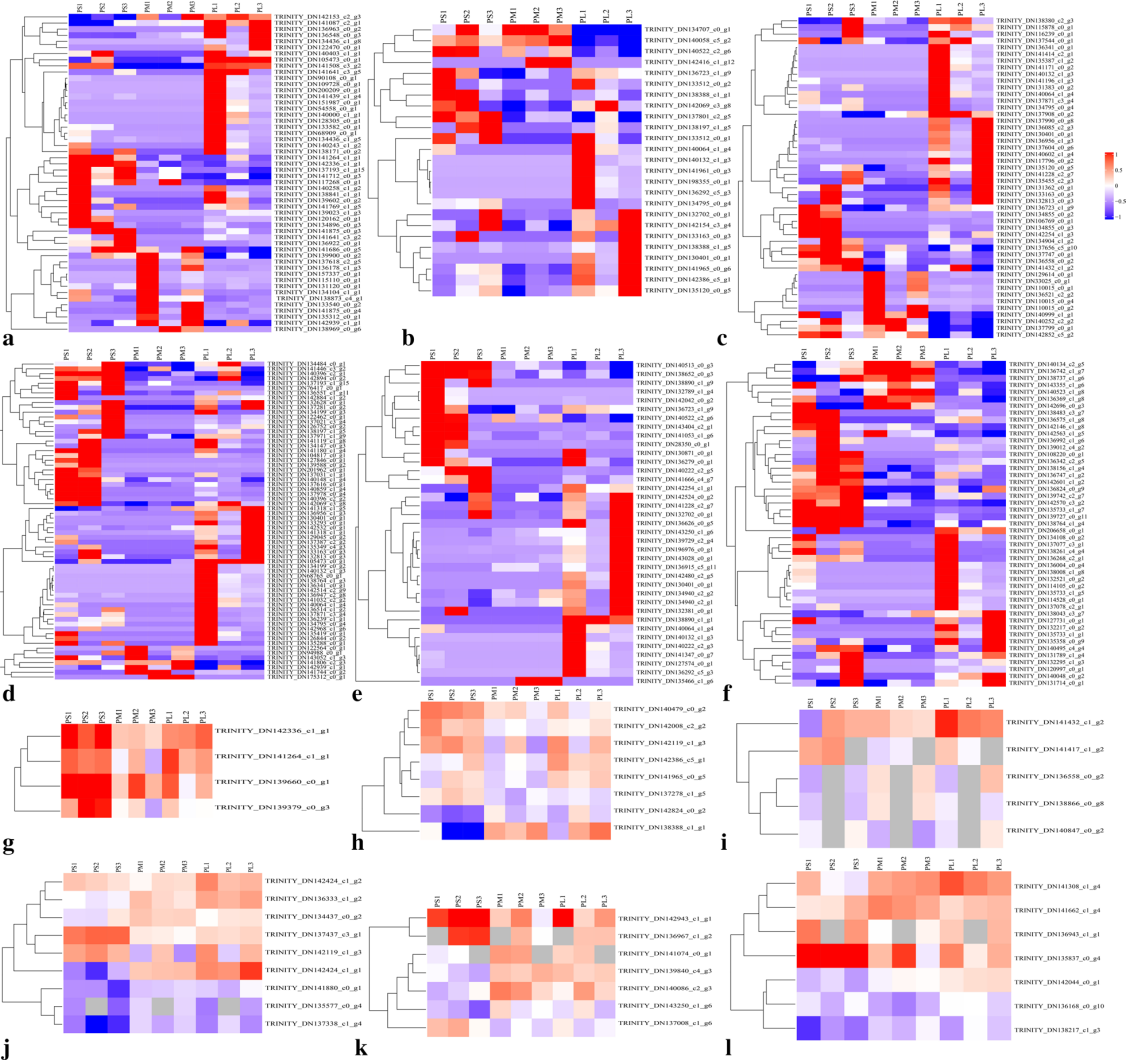
Heatmap analysis of DEGs and DAPs based on transcriptomic and proteomic, which are associated with cell wall metabolic processes. Red indicates significantly upregulated proteins, and blue indicates significantly downregulated proteins. White indicates proteins with no significant changes. **a**, **g** Heat map of phenylpropanoid biosynthesis-associated gene and protein expression. **b**, **h** Heat map of galactose metabolism-associated gene and protein expression. **c**, **i** Heat map of amino sugar and nucleotide sugar metabolism-associated gene and protein expression. **d**, **j** Heat map of starch and sucrose metabolism-associated protein expression. **e**, **k** Pentose and glucuronate interconversions metabolism-associated gene and protein expression. **f** Cell wall-related transcription factor. **l** Cell wall metabolism-associated protein expression

KEGG pathway analysis indicated that many DEGs were enriched in metabolic pathways and ribosomes in the PM_PS comparison and in the biosynthesis of secondary metabolites and metabolic pathways in the PL_PM comparison (Fig. 2e, f). Cluster analysis showed that cell wall-related DEGs (285), including pentose and glucuronate interconversion, the phenylpropanoid pathway, galactose metabolism, starch and sucrose metabolism, amino sugar and nucleotide sugar metabolism, and transcription factors, were clustered closely in the PM_PS and PL_PM groups. Most DEGs were downregulated in the PM_PS group, but these were upregulated in the PL_PM group (Fig. 3).

### Quantitative proteome analysis

In total, 812,625 spectra, 68,151 identified spectra, 12,456 peptides, 10,572 unique peptides, and 2839 proteins were determined via proteomic analysis (Table 1 and Additional file 1: Table S3). In terms of protein mass distribution, proteins with molecular weights greater than 9 kDa had a wide range and good coverage, with a maximum distribution area of 10–40 kDa. Peptide quantitative analysis of the proteins showed that protein quantity decreased with an increase in matching peptides (Additional file 1: Fig. S3).

Among these 2839 proteins, 223 were identified as differentially abundant proteins (DAPs), including 173 (75 up- and 98 downregulated), 33 (20 up- and 13 downregulated), and 17 co-expressed proteins in the PM_PS and PL_PM groups, respectively (Table 2), which are presented in a volcano plot (Additional file 1: Fig. S2c, d). The summary information of these DAPs is shown in Additional file 1: Table S4.

### Functional classification of the identified DAPs

GO analysis showed that most DAPs in the BP category were involved in cellular responses to chemical stimulus and cellular oxidant detoxification processes in the PM_PS group and in metabolic processes and macromolecule metabolic processes in the PL_PM group. The highest proportions of DAPs in the MF category were involved in oxidoreductase activity and antioxidant activity in the PM_PS group and structural constituent of ribosome and structural molecule activity in the PL_PM group. The extracellular region in the PM_PS group and cytoplasmic parts in the PL _PM group showed the highest portions of DAPs in the CC category (Fig. 2c, d). KEGG pathway analyses indicated that many proteins were enriched in the two-component system and ribosome pathways in the PM_PS and PL_PM groups, respectively (Fig. 2g, h).

**Fig. 4:**
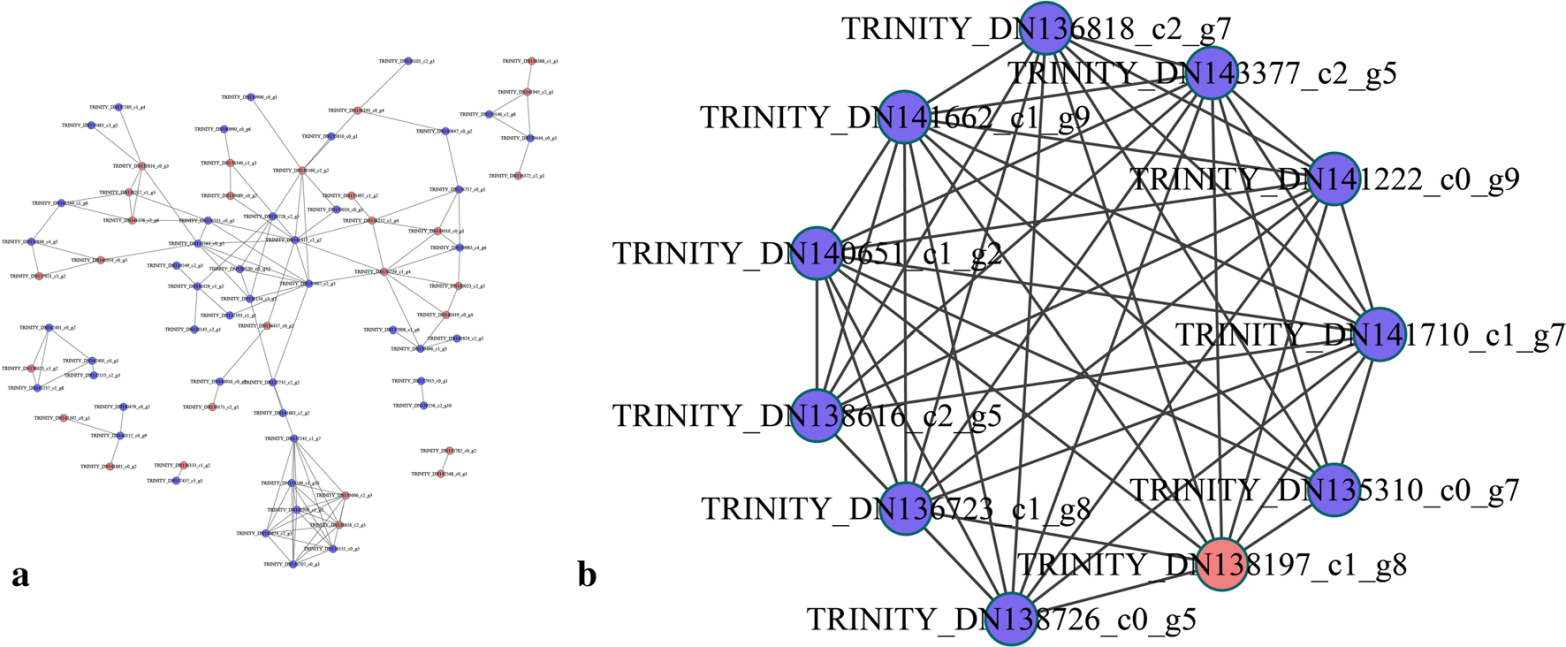
Analysis of the functional network by STRING 9.0 of DAPs. **a** Analysis of the functional network by STRING 9.0 of DAPs in PM_PS. **b** Analysis of the functional network by STRING 9.0 of DAPs in PL_PM. Red indicates significantly upregulated proteins, and blue indicates significantly downregulated proteins

Hierarchical cluster analysis showed that 40 cell wall-related DAPs, including pentose and glucuronate interconversions, the phenylpropanoid pathway, galactose metabolism, starch and sucrose metabolism, amino sugar and nucleotide sugar metabolism, and other cell wall metabolism-related proteins, were clustered closely in the PM_PS and PL_PM groups. Notably, most DAPs were upregulated in both the PM_PS and PL_PM groups. Alternatively, DAPs involved in phenylpropanoid pathways and galactose metabolism were downregulated in the PM_PS group but upregulated in the PL_PM group (Fig. 3g–l). Moreover, the protein–protein interaction (PPI) network analysis indicated that 85 DAPs, including 29 upregulated (28 and 1) and 56 downregulated DAPs (46 and 10), were involved in the interaction networks in the PM_PS and PL_PM groups, respectively (Fig. 4).

### Comparative analysis between protein abundance and gene expression

**Fig. 5:**
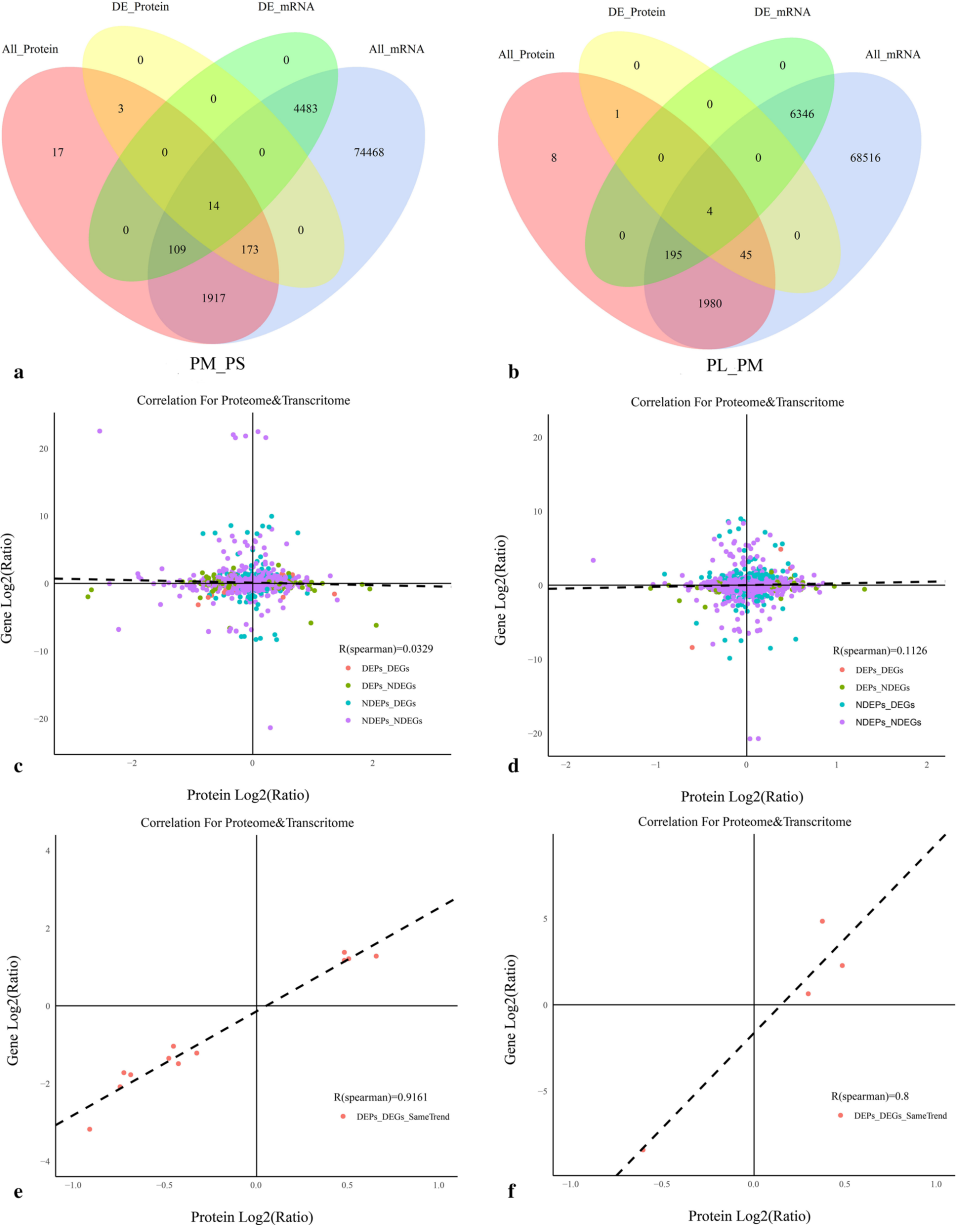
Correlations between mRNA and protein expression. **a** Venn diagram of genes quantified in the transcriptome (blue) and proteome (pink), DEGs(green) and DAPs (yellow) in PM_PS. **b** Venn diagram of genes quantified in the transcriptome (blue) and proteome (pink), DEGs(green) and DAPs (yellow) in PL_PM. **c** Scatterplot of the relationship between genes identified in both the transcriptome and proteome in PM_PS. **d** Scatterplot and correlation coefficients between DEGs and DEPs in PL_PM. **e** Scatterplot and correlation coefficients between DEGs and DEPs (the same trend) in PM_PS. **f** Scatterplot and correlation coefficients between DEGs and DEPs (the same trend) in PL_PM

There were more DEGs (11,205) than DAPs (240) and shared DEGs (1904) than shared DAPs (17) in cracked fruit than in non-cracking fruit. Most were downregulated in the PM_PS group but upregulated in the PL_PM group (Table 2). Moreover, integration of the proteome and transcriptome data showed that 14 and 4 DAPs were matched with their DEGs. In addition, 12 (4 up- and 8 downregulated) and 4 DAPs (2 up- and 2 downregulated) showed the same tendency as DEGs in the PM_PS and PL_PM groups, respectively, with two showing the opposite tendency as DEGs in the PM_PS group (Fig. 5a, b). Furthermore, the fold-changes in DAPs indicated differentially positive correlations with their corresponding DEGs based on Pearson’s correlation tests. A limited correlation (*r *= 0.03 and 0.11) was detected between the proteome and transcriptome, and a relatively higher positive correlation was identified with the same trend (*r* = 0.9161 and 0.8) for DEGs and DAPs in the PM_PS and PL_PM groups, respectively (Fig. 5c–f).

### Identification of DAPs and DEGs associated with candidate pathways

The correlation of proteome and transcriptome GO enrichment indicated that the largest groups of those DEGs and DAPs within the BP, MF, and CC categories were linked to metabolic and cellular processes, catalytic activity and binding, and cells and cell parts, respectively, in both the PM_PS and PL_PM groups (Additional file 1: Fig. S4a–b). KEGG enrichment showed that most were significantly enriched in phenylpropanoid biosynthesis, pentose and glucuronate interconversions, amino sugar and nucleotide sugar metabolism, and galactose metabolism in the PM_PS group. Most were significantly enriched in pentose and glucuronate interconversions and the galactose metabolism pathway in the PL_PM group for both the proteome and transcriptome (Additional file 1: Fig S4c–d). Comparative analysis showed that two pathways, i.e., pentose and glucuronate interconversion and galactose metabolism pathways, were shared in both the PM_PS and PL_PM groups. In contrast, the phenylpropanoid biosynthesis pathway was only shared in the PM_PS group.

### Validation of data reliability through reverse transcription real-time quantitative PCR (qPCR) and parallel reaction monitoring (PRM)

**Fig. 6:**
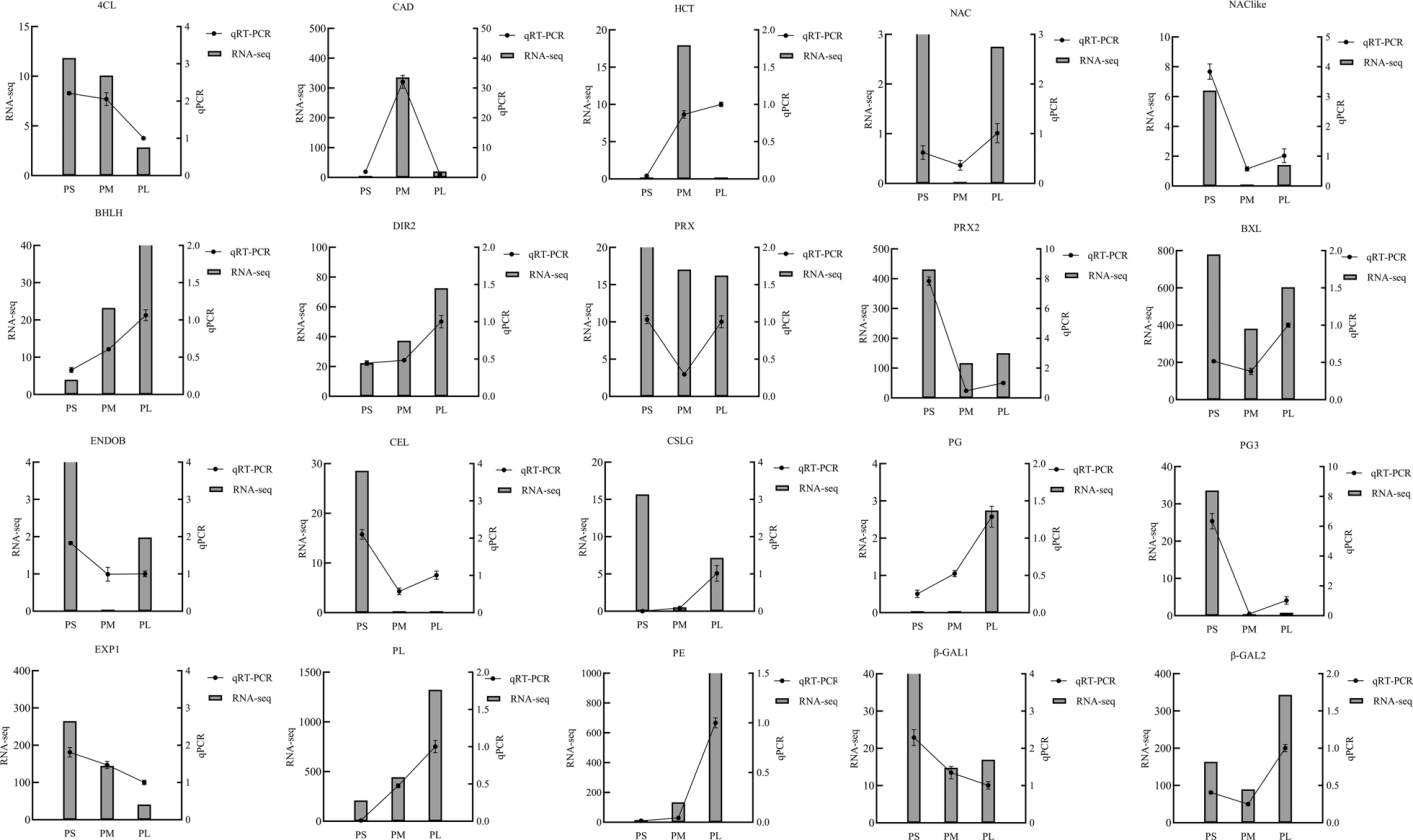
Validation and expression analysis of selected genes using qPCR. The expression levels of the genes revealed by RNA-seq (Left y-axis) and qPCR (right y-axis). Histograms were gene expression detected by RNA-seq. Line graphs were relative expression validated by qPCR

qPCR experiments were performed for 20 selected DEGs, and the results are shown in Fig. 6. In the PM_PS group, the phenylpropanoid pathway-related genes 4-coumarate-COA-ligase (*4CL*), peroxidase (*PRX*), and *PRX2* were downregulated, whereas cinnamyl-alcohol dehydrogenase (*CAD*) and shikimate O-hydroxycinnamoyltransferase (*HCT*) were upregulated. The galactose metabolism-related genes *β-GAL1* and *β-GAL2*; amino sugar and nucleotide sugar metabolism-related gene beta-d-xylosidase (*BXL*); and starch and sucrose metabolism-related genes cellulase (*CEL*), cellulose synthase-like protein (*CSLG*), and glucan endo-1,3-beta-d-glucosidase (*ENDOB*) were downregulated. The cell wall metabolism genes *NAC*, *NAC*-like, and *EXP1* were downregulated, whereas the *BHLH* transcription factor and dirigent protein (*DIR2*) were upregulated. The pentose and glucuronate interconversion-related genes *PL*, *PG*, and *PE* were upregulated. Most of these genes, except *4CL*, *CAD*, *β-GAL*, and *EXP1*, were significantly upregulated in the PL_PM group. Moreover, the expression of 13 candidate genes, including *DIR2*, *NAC-*like, *EXP1*, *CAD*, *β-GAL1*, *β-GAL2*, *4CL*, *ENDOB*, *PE*, *BHLH*, *PG3*, *CEL*, and *PRX2*, showed strong correlations and that of seven genes showed poor correlations with the corresponding RNA-seq data (Fig. 6; Table 3).

**Table 3:** Correlation analysis between qPCR and FPKM values of selected genes

Accession	Gene description	Gene name	Pearson correlation efficient	*P *value	Numbers
TRINITY_DN135342_c3_g3	Dirigent protein 22-like	DIR2	0.7977	0.0100	18
TRINITY_DN138969_c0_g6	Shikimate O- hydroxycinnamoyltransferase	HCT	0.2848	0.4577	18
TRINITY_DN136551_c1_g11	Cellulose synthase-like protein G3	CSLG	− 0.06589	0.8663	18
TRINITY_DN136342_c2_g5	NAC domain-containing protein 100-like	NAC-like	0.9464	0.0001	18
TRINITY_DN141308_c1_g4	Expansin-A1	EXP1	0.8582	0.0031	18
TRINITY_DN141875_c0_g4	Cinnamyl alcohol dehydrogenase	CAD	0.8015	0.0094	18
TRINITY_DN138388_c1_g1	Beta-galactosidase 3-like	β-GAL1	0.8440	0.0169	18
TRINITY_DN142386_c5_g1	Beta-galactosidase	β-GAL2	0.6675	0.0495	18
TRINITY_DN196976_c0_g1	Polygalacturonase	PG	0.7863	0.0636	18
TRINITY_DN141686_c0_g5	4-coumarate–CoA ligase-like 5	4CL	0.7466	0.0208	18
TRINITY_DN138197_c1_g5	Glucan endo-1,3-beta-glucosidase	ENDOB	0.7052	0.0338	18
TRINITY_DN131789_c1_g4	NAC domain-containing protein	NAC	0.1755	0.6515	18
TRINITY_DN143028_c0_g1	Pectinesterase	PE	0.8042	0.0292	18
TRINITY_DN142336_c1_g1	Peroxidase	PRX	0.4798	0.1912	18
TRINITY_DN141432_c1_g2	Beta-D-xylosidase 2	BXL	0.1761	0.6505	18
TRINITY_DN143250_c1_g6	Pectate lyase	PL	0.6021	0.0862	18
TRINITY_DN138043_c3_g7	Transcription factor bHLH66	BHLH	0.7485	0.0327	18
TRINITY_DN142042_c0_g2	Polygalacturonase	PG3	0.6819	0.0430	18
TRINITY_DN76417_c0_g1	Glucan 1,3-beta- glucosidase	CEL	0.8732	0.0103	18
TRINITY_DN141264_c1_g1	Peroxidase N1	PRX2	0.8325	0.0054	18

**Fig. 7:**
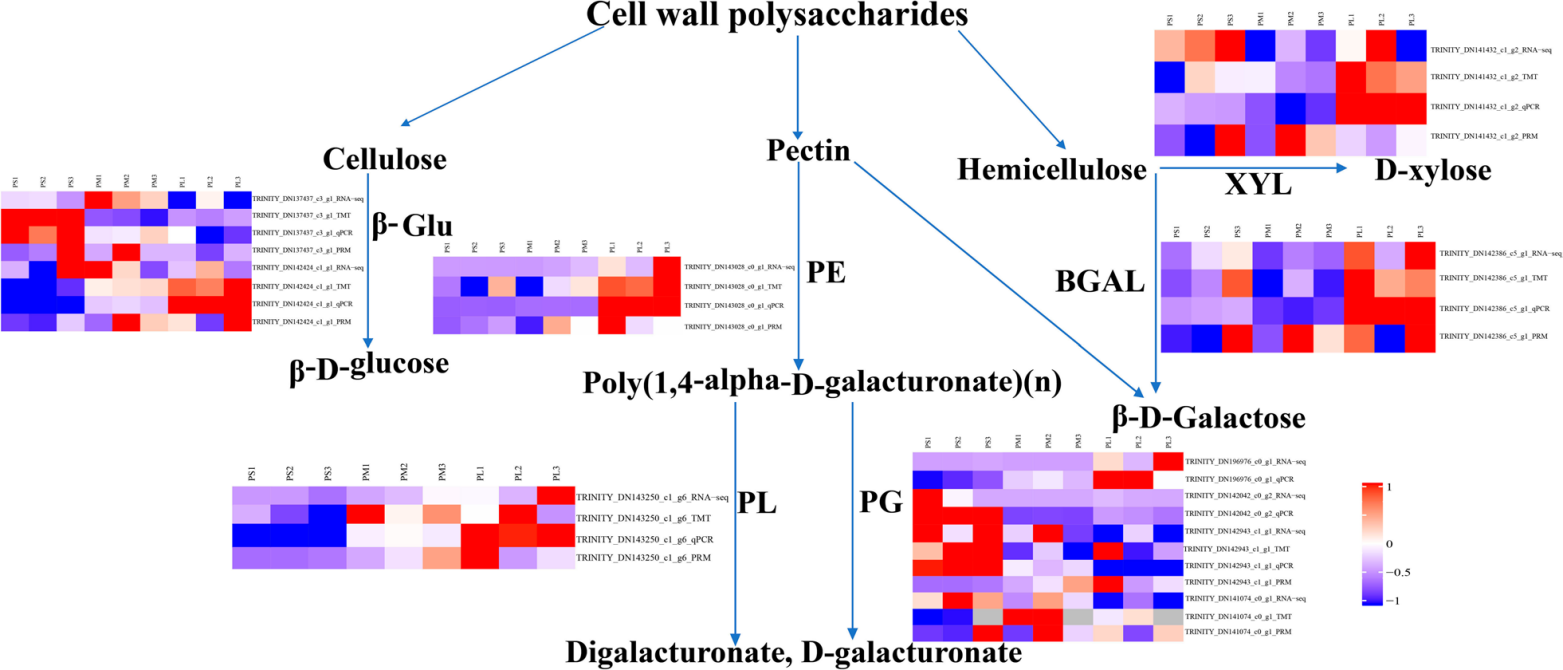
Summary of some of the biological pathways involved in *A. trifoliate* fruit ripening and cracking. TRINITY_DN143250_c1_g6 (PL); TRINITY_DN196976_c0_g1 (PG), TRINITY_DN142042_c0_ g2 (PG3); TRINITY_DN142943_c1_g1 (PG2); TRINITY_DN143028_c0_g1 (PE); TRINITY_ DN141074_c0_g1 (PE2); TRINITY_DN137437_c3_g1 (BGLU33); TRINITY_DN141880_c0_g1 (ENBG); TRINITY_DN142424_c1_g1 (F26G); TRINITY_DN141432_c1_g2 (BXL). Red indicates significantly upregulated proteins, and blue indicates significantly downregulated proteins. White indicates proteins with no significant changes

Moreover, 20 DAPs were selected for PRM analysis, of which 18 exhibited significantly different levels. Of these 18 DAPs, 14 (77.8%) showed the same trends in abundance between PRM and TMT quantification, including *PE*, *PL*, *PG2*, furostanol glycoside 26-O-beta-glucosidase (*F26G*), *β-GAL2*, auxin efflux carrier, alpha/beta hydrolase (*α-HY*), *PRX2*, *PG4*, *PRX5*, *PRX3*, endoglucanase 19, endoglucanase 8, and *DIR1*. Additionally, four genes (*PRX*, beta-fructofuranosidase, *BXL*, and *BGLU33*) showed inconsistent abundance compared with protein levels quantified by TMT (Table 4). In general, the trends in the expression changes measured by PRM and TMT were consistent. Notably, in the PM_PS and PL_PM groups, several genes involved in cell wall metabolism pathways showed consistent upregulation/downregulation in the transcriptome and proteome (Fig. 7).

**Table 4:** Comparison of PRM and TMT quantification results

Accession	Protein description	Gene name	PM_PS ratio		PL_PM ratio	
			PRM	TMT	PRM	TMT
TRINITY_DN143250_c1_g6	Pectate lyase	PL	43.325	1.392	2.029	#N/A
TRINITY_DN143028_c0_g1	Pectinesterase	PE	1.470	0	1.695	1.293
TRINITY_DN142943_c1_g1	Polygalacturonase	PG2	0.799	0.566	0.993	#N/A
TRINITY_DN142424_c1_g1	Furostanol glycoside 26- O-beta-glucosidase	F26G	4.390	1.853	1.185	1.301
TRINITY_DN142386_c5_g1	Beta-galactosidase	β-GAL2	1.620	0	1.071	1.394
TRINITY_DN142336_c1_g1	Peroxidase	PRX	1.303	0.594	0.707	1.304
TRINITY_DN142120_c0_g1	Auxin efflux carrier	AEC	2.593	1.245	0.535	0
TRINITY_DN142119_c1_g3	Beta-fructofuranosidase	β-FRU	1.497	0.655	0.500	0
TRINITY_DN141662_c1_g4	Alpha/beta hydrolase	α-HY	3.192	1.243	0.306	0
TRINITY_DN141432_c1_g2	Beta-D-xylosidase	XYL	1.717	0	0.606	1.398
TRINITY_DN141264_c1_g1	Peroxidase N1	PRX2	0.828	0.619	1.651	0
TRINITY_DN141074_c0_g1	Polygalacturonase	PG4	1.620	1.464	0.680	0
TRINITY_DN139660_c0_g1	Peroxidase 53	PRX5	0.703	0.644	1.239	0
TRINITY_DN139379_c0_g3	Cationic peroxidase 1	PRX3	0.421	0.546	1.208	0
TRINITY_DN137437_c3_g1	Beta-glucosidase 33	BGLU33	1.291	0.636	0.317	0
TRINITY_DN137338_c1_g4	Endoglucanase 19	ENDOG19	15.470	1.502	0.232	0
TRINITY_DN136333_c1_g2	Endoglucanase 8	ENDOG8	3.207	1.220	0.696	0
TRINITY_DN135837_c0_g4	Dirigent-like protein	DIR1	0.733	0.597	0.927	0

## Discussion

### Structural changes in the pericarp cell wall might affect *A. trifoliata* fruit cracking

Fruit cracking occurs when the stress exerted on the pericarp from the enlarged aril is greater than the strength of the fruit skin, and the mechanical strength of the pericarp depends largely on its cell wall [29]. Studies have shown that jujube fruit cracking may be related to structural changes and rearrangement of the cell wall during the later stages of fruit ripening [30]. Arrangement of the subcutaneous layers of cells was found to be relatively regular, and cell layers had a closer arrangement in the cracking-resistant tomato genotype [31]. In this study, the cell wall of the pericarp had poor integrity, loose structures, deformed and reduced cell layers, and larger spaces and began to degrade in cracked fruits during ripening, consistent with previous results in grapes and oranges [32, 33]. This suggests that structural changes in the cell wall might play key roles in the occurrence of *A. trifoliata* fruit cracking.

### General features of the transcriptomes and proteomes of different *A. trifoliata* pericarps

Fruit cracking is a key factor affecting seed yield. Elucidating the molecular mechanisms underlying fruit cracking could aid the utilization of *A. trifoliata* seeds for biofuels. However, the underlying mechanisms of *A. trifoliata* fruit cracking remain largely unknown. In this study, differences in the transcriptome and proteome were investigated based on RNA-seq and TMT data during different stages. As the transcriptome database was used for protein identification, the quality of the sequencing and assembly of the transcriptome data was crucial for subsequent analyses. In total, 186,054 unigenes (> 200 bp) were assembled in the transcriptome of *A. trifoliata* pericarp, and this number is much higher than that previously obtained for this species and other Ranunculales spp., e.g., *A. trifoliata* (11,749 by Yang et al. [26]; 65,757 by Niu et al. [1]), *Dysosma aurantiocaulis* (53,929) [34], and *D. versipellis* (44,855) [35]. The obtained percentages of the Q30 bases (91.58–93.75%) and GC contents (45.94–48.48%) were similar to those reported in other studies of the *A. trifoliata* transcriptome, e.g., 89.06–93.33% and 43.20–43.93% [26] and 96.31% and 45.10% [1], respectively. In total, 100,329 (53.9%) and 56,346 (30.3%) of all unigenes identified in the current study matched those in the NR and SwissProt databases, respectively. These results are similar to values reported for *D. aurantiocaulis* (29,497 and 18,029; 54.70% and 33.43%) [34] and were higher than those presented in other similar *A. trifoliata* transcriptome studies, including 34,245 and 23,352 (32.14% and 21.91%) in the NR and SwissProt databases, respectively [26], and 19,096 (29.95%) in SwissProt [1]. Thus, our results provided extensive sequence and unigene resources for *A. trifoliata*.

Moreover, 812,625 spectra, 10,572 unique peptides, and 2839 proteins were identified based on transcriptome data from *A. trifoliata* pericarp. The RNA-seq and protein sequencing methods identified and annotated many genes and proteins, providing a basis for a more precise and detailed descriptions of molecular processes and for the elucidation of complex physiological processes and their genetic regulation [36]. The data presented here were sufficiently high and provided useful tools for future genetic research on fruit cracking in *A. trifoliata* and other Lardizabalaceae species. Additionally, there were many “uncharacterized,” “predicted,” or “putative” transcripts and proteins in the annotation results, indicating the limited nature of the analysis owing to a lack of genomic information [37]. Thus, the roles of these unknown or uncharacterized genes and proteins in *A. trifoliata* fruit cracking should be investigated in future studies.

### Translational and post-translational regulation of *A. trifoliata* fruit cracking

Some inevitable passive processes will occur in the cracked pericarp following fruit cracking, such as oxidative stress and microbial invasion [27]. Therefore, differences in the expression of genes and proteins among PS, PM, and PL alone cannot accurately reflect the cause of fruit cracking. In this study, our comparative analysis of protein and gene expression levels indicated that more DEGs and shared DEGs than DAPs were identified in cracked fruits than in non-cracking fruits. This could be explained by the technical limitations of MS-based proteomics, such as low amount of readily available samples and low MS scanning rate for quantitative data acquisition, and the need for extensive fractionation, limiting the capacity to identify proteins [38]. The PPI analysis indicated that the interactions of proteins were infrequent and weak, which was associated with the results that the interactions are often weak for many cellular processes, which are regulated by post-translational modifications that are recognized by specific domains in protein binding partners [39]. Moreover, correlation analyses showed negative correlations between the proteome and transcriptome, indicating a discordance between the transcript levels and protein abundance. These results were similar to those observed in previous reports, suggesting that post-transcriptional and post-translational regulation, reversible phosphorylation, splicing events in cells, and translation efficiency play key roles in the regulation of fruit ripening [40, 41]. Thus, gene translation and post-translation processes could be important regulatory methods in fruit ripening and cracking.

### Potential regulators and metabolic pathways during fruit cracking

GO and KEGG functional enrichment provide prediction information of inner-cell metabolic pathways and of the genetic and biologic behaviors of genes. GO terms associated with oxidoreductase activity and structural molecule activity were mainly enriched in the PM_PS and PL_PM groups, respectively, similar to previous studies showing that the target genes could be classified into different categories based on their functions, such as peroxidase and cell wall polysaccharide [20, 42]. Cell polysaccharides are degraded by cell wall hydrolases, and whereas phenolic crosslinking of cell wall structural components is catalyzed by cell wall peroxidase. These modifications reduce the strength of the fruit pericarp, resulting in changes in pericarp development and fruit cracking [43]. KEGG analysis indicated that cell wall-related pathways, including pentose and glucuronate interconversions, the galactose metabolism pathway, and the phenylpropanoid biosynthesis pathway, were common pathways shared by DEGs and DAPs, suggesting that cell well metabolism may have important roles in *A. trifoliata* fruit ripening and cracking. Moreover, these results revealed that the proteomic data and transcriptome data were complementary, and that the proteome could confirm the transcriptome data; in addition, genes perform the same function at the transcriptome and proteome levels [44]. Functional classification of the transcriptome and proteome could improve our understanding of the molecular physiology of fruit ripening and cracking.

### Candidate DEGs and DAPs may play key roles in fruit pericarp cracking

Fruit cracking is a complex physiological process that is controlled by numerous genes working together, rather than a single gene directly controlling the process [18, 45]. Researchers have found that several cell wall modification genes, including *β-GAL*, *β-GLU*, *PE* and *PG* are differentially expressed in cracked fruits compared with levels in non-cracking litchi fruits [21]. Downregulation of *PpBGAL* can delay peach fruit softening by reducing PG and pectin methylesterase activity [46]. Antisense inhibition of PE and PG activity in tomatoes was found to reduce fruit cracking [47]. Additionally, silencing the *SIPL* gene can enhance fruit firmness and reduce the content of pectin, suggesting that this gene participates in pericarp cell wall rearrangement during fruit softening [48]. Fruit softening is mainly caused by hemicellulose and pectin degradation proteins, such as XYL, BGAL, PE, and PG [49, 50]. As a major cellulose degradation enzyme, downregulation of BGLU protein in strawberries can delay fruit maturation [51]. PRXs are also involved in rearrangement of cell wall polysaccharides during plant development [52].

In this study, 13 DEGs and 14 DAPs involved in cell wall metabolism showed strong correlations with RNA-seq data and protein expression levels, consistent with results showing that cell polysaccharide metabolism might play key roles in fruit ripening and cracking [42, 43]. Notably, the expression levels of three proteins (β-GAL2, PE, and PL) associated with galactose metabolism and the pentose and glucuronate interconversion pathways showed higher protein and gene expression in cracking fruit than in non-cracking fruit, suggesting that PE, PL, and β-GAL2 may play important roles in the regulation of *A. trifoliata* fruit cracking.

In addition, F26G, PG, PG3, XYL, PRX3, and PRX5 also showed higher protein and gene expression in cracking fruit than in non-cracking fruit. The significantly increased expression of these cell wall metabolism proteins indicated a network of cell wall cellulose, hemicellulose, and pectin in fruit cracking during ripening [50, 53].

## Conclusions

Our findings reveal the structural changes in the cell wall during different stages of *A. trifoliata* pericarp development, suggesting that structural changes between unripe and ripe fruits are important factors affecting the cracking tendency of fruits. To strengthen the structure of the pericarp, bagging and 1-MCP treatment, which reduce fruit cracking, could be performed [54, 55]. Comparative transcriptomic analysis and proteomic profiling were applied to identify the potential genes/proteins that participated in fruit cracking. Various genes and proteins were found to be differentially expressed after cracking. Three co-expressed genes (*PE*, *PL*, and *β-GAL2*) were implicated in various signaling pathways in fruit development, cell wall degradation, and fruit softening. These omics data provide a new perspective to understand the process of fruit cracking in *A. trifoliate*. In future works, transgenic plants overexpressing candidate genes are needed to validate the functions of these genes. Molecular markers for fruit cracking will be developed to utilize this trait in breeding and to increase seed yields for biorefineries.

## Methods

### Plant materials

The wild germplasm Nong No. 8, which had been transplanted 9 years prior in the nursery at Hunan Academy of Agricultural Sciences, Changsha, P. R. China, was used as the research material in this study. According to our observations, in Changsha, Hunan Province, the blooming stage for the germplasm of Nong No. 8 was in early April, when 50% of the *A. trifoliata* flowers were in bloom with a flowering period of approximately 30–45 days. Fruit development lasts for 5 months, from ovary inflation through fruit setting, and longitudinal and transverse elongation, flesh softening, and peel cracking complete the development process [56]. The Nong No. 8 variety usually ripens in early October. Because the fruit of *A. trifoliata* do not develop uniformly, fruits were harvested from different stages of the same Nong No. 8 tree at the same time and were then sorted according to their developmental stage, considered to be a sample. Different fruits, including non-cracking fruits (PS), initially cracking fruits (PM), and total cracking fruits (PL) were randomly collected every 10 days during the ripening stage (September 18, September 28, and October 8, 2018) and three fruits were mixed into one biological replicate for further analysis. In total, three biological replicates were collected for each stage. Sampled pericarps were rapidly collected and immediately frozen in liquid nitrogen and stored at − 80 °C until use for transcriptome, proteome, qPCR, and PRM analyses.

### Anatomical structure of the pericarp

The anatomy of pericarp samples collected from the Nong No. 8 fruits at different development stages, including PS, PM, and PL, were prepared for paraffin sectioning, and scanning electron microscopy (SEM) was carried out according to previous studies [57, 58]. Pericarp samples were fixed directly in the field using FAA (70% ethyl alcohol + 38% methyl aldehyde + 25% acetic acid [16:1:1]). The tissues were subsequently dehydrated through an ethanol series with increasing ethanol concentrations and embedded in paraffin. Subsequently, paraffin sections were stained with Safranin O and Fast Green staining and observed with an Axio Imager (Zeiss, Oberkochen, Germany); upright microscopy images were displayed using Image-pro Plus 6.0 software.

Pericarp samples were fixed in 2.5% glutaraldehyde (pH 7.4) for 4 h under syringe suction and washed subsequently in phosphate buffered-saline (PBS, 0.1 M, pH 7.0). After post-fixing with 1% osmium for 1–2 h and washing three times in PBS, samples were dehydrated with 30%, 50%, and 70% ethanol for 20 min and then with ethanol and iso-amyl acetate (V:V = 1:1) for 30 min. Samples were finally dried in liquid carbon dioxide. Dried samples were coated with gold–palladium in a Hitachi Model E-1010 ion sputter for 4–5 min and observed via SEM with a Hitachi Model SU-8010 (Hitachi, SU-8010, Japan).

### RNA isolation, library construction, and sequencing

Total RNA used for the RNA-seq assays was isolated from three independent replicates of pericarp in the PS, PM, and PL stages, as described by Tao et al. [59]. RNA samples were detected based on the A260/A280 absorbance ratio with a Nanodrop ND-1000 system (Thermo Scientific, USA). Paired-end libraries were prepared using a NEBNext UltraTM RNA Library Prep Kit for Illumina (NEB, USA) following the manufacturer’s instructions. The mRNA was purified from 3 µg total RNA using oligo (dT) magnetic beads followed by fragmentation carried out using divalent cations at elevated temperatures in NEBNext First Strand Synthesis Reaction Buffer. Subsequently, first-strand cDNAs were synthesized with random hexamer primers and Reverse Transcriptase (RNase H-) using mRNA fragments as templates, followed by second-strand cDNA synthesis using DNA polymerase I, RNAseH, buffer, and dNTPs. The synthesized double-stranded cDNA fragments were then purified with an AMPure XP system (Beckman Coulter, Beverly, MA, USA). The purified double-stranded cDNA was polyadenylated and adapter-ligated for preparation of the paired-end library. Adaptor-ligated cDNA and adaptor primers were used for PCR amplification. PCR products were purified (AMPure XP system) and library quality was assessed on an Agilent Bioanalyzer 2100 system. Finally, sequencing was performed with an Illumina HiSeq2500 instrument by Shanghai Applied Protein Technology (Shanghai, China).

### Quality control and transcriptome assembly

The raw paired-end reads in fastq format produced from the sequencing were first processed with the in-house Perl scripts. Reads containing adapters, excess “N” nucleotides with more than 10% of the bases and reads of low-quality (reads with quality values ≤ 10) were removed by filter_fq software. The Q20, Q30, GC content, and sequence duplication levels of the obtained clean reads were calculated. The assembly of clean reads to unigene collections was performed using the Trinity software package (https://github.com/trinityrnaseq/trinityrnaseq/releases) [60]. Trinity software consists of three independent software modules, including Inchworm, Chrysalis, and Butterfly, and transcripts less than 200 bp in length were discarded. Sequences containing the longest cluster transcripts without redundancy extracted from transcripts were considered unigenes.

### Bioinformatics analyses

The de novo assembled unigenes were annotated in five databases, including NR, Pfam, SwissProt, GO, and KEGG pathway databases, based on a BLAST search with an E-value threshold of 1E^−5^. Moreover, to further analyze the annotation results, GO and KEGG results with E-values of 1E^−5^ were used for functional gene annotation. GO terms could be classified into three categories, including BP, MF, and CC. In addition to the GO terms, the pathway maps were determined based on the KEGG database.

The normalized transcript abundances of the genes were estimated using the fragments per kilobase per million reads mapped based on the length of the gene and read counts mapped to this gene. DESeq2 R package (1.16.1) software was used to identify DEGs, and the false discovery rate (FDR) was controlled using the Benjamini and Hochberg’s approach to adjust the *P* value. Genes with an adjusted *P* value of less than 0.05 and absolute fold-change of 2 were deemed to be differentially expressed between the two samples. In addition, GO and KEGG pathway enrichment analysis of DEGs was implemented with the clusterProfiler R package. Transcription factor analysis of DEGs was performed against the PlantTFDB database (https://planttfdb.cbi.pku.edu.cn/). The heat map was visualized using heatmap 2.0 in the gplot R package.

### Protein extraction

Protein extraction from *A. trifoliata* pericarp was performed from each sample as described previously [61]. The samples were frozen in liquid nitrogen and ground into powder. A five-fold volume of TCA/acetone (1:9) was added, and the sample was vortexed, mixed, and incubated at − 20℃ for 4 h. Samples were then then centrifuged at 6000×*g* at 4℃ for 40 min. The supernatant was discarded, and the precipitate was washed three times with pre-cooled acetone. After precipitation and air drying, the precipitate was redissolved in buffer (4% sodium dodecyl sulfate [SDS], 100 mM Tris–HCl and 1 mM DTT; pH 7.6). After sonication and boiling for 15 min, the lysate was centrifuged for 40 min, and the supernatant was filtered and quantified using the BCA Protein Assay Kit (Bio-Rad, USA).

### Trypsin digestion and TMT labeling

For digestion, the samples were added to the buffer (4% SDS, 100 mM DTT, 150 mM tris–HCl, pH 8.0) and UA buffer (8 M urea, 150 mM Tris–HCl pH 8.0) by repeated ultrafiltration (Microcon units, 10 kDa). Then, iodoacetamide (100 mM IAA in UA buffer) was added to the samples to block reduced cysteine residues, and samples were incubated for 30 min in the dark at room temperature. After the filters were washed with UA buffer and triethylamine borane (TEAB) buffer in turn, the suspensions were digested with trypsin (Promega, Madison, WI, USA) in TEAB buffer overnight. After trypsin digestion, the samples (100 μg protein) were categorized for labeling with 129-tag (PS), 130-tag (PM), and 131-tag (PL; Thermo Fisher Scientific, Waltham, MA, USA). Finally, TMT-labeled peptide aliquots were pooled for subsequent fractionation using a Pierce high pH reversed-phase fractionation kit (Thermo scientific, USA).

### High-performance liquid chromatography fractionation and liquid chromatography-tandem mass spectrometry (LC–MS/MS) analysis

For the fractionation of labeled peptides, samples were loaded onto a reverse-phase trap column (Thermo Scientific Acclaim PepMap100, 100 μm × 2 cm, nanoViper C18) connected to the C18 reverse-phase analytical column (Thermo Scientific Easy Column, 10 cm long, 75 μm inner diameter, 3 μm resin) in buffer A (0.1% formic acid) and separated with a linear gradient of buffer B (84% acetonitrile and 0.1% formic acid) at a flow rate of 300 nL/min controlled by IntelliFlow technology. The resulting peptides were further processed using a Q Exactive mass spectrometer (Thermo Scientific, USA) coupled with an Easy nLC (Thermo Fisher Scientific). MS analysis was performed in positive ion mode, and MS data were acquired using a data-dependent top10 method dynamically choosing the most abundant precursor ions from the survey scan (300–1800 m/z) using the higher-energy collision dissociation (HCD) fragmentation method. Automatic gain control (AGC) was set as 3E6 and the maximum inject time was 10 ms, with the following parameters: 40.0-s dynamic exclusion duration; 70,000 resolutions with survey scans at m/z 200 and resolution for HCD spectra at 17,500 at m/z 200; 2 m/z of isolation width; 30 eV of normalized collision energy and the underfill ratio was defined as 0.1%.

### Sequence database search and data analysis

The raw data were processed by the MASCOT engine (Matrix Science, London, UK; version 2.2), and Proteome Discoverer 1.4 software was used to process MS/MS spectra. The search was performed using the following settings based on the *A. trifoliata* database: trypsin for the enzyme and 2 as the maximum missed cleavage allowed; fixed modifications of carbamidomethyl (C), TMT-6plex (N-term), and TMT-6plex (K), variable modification of oxidation (M); mass tolerance for fragment ions of 0.1 Da, and 20 ppm for peptide ions, as well as both peptide and protein levels of FDR less than 0.01, and only unique peptides of the protein were employed for the protein identification and quantification. Proteins with a *P* value less than 0.05 and fold-change greater than or equal to 1.2 or less than or equal to 0.83 within a comparison were recognized as DAPs.

Functional categorization was performed using GO and KEGG pathway databases with *P* values less than or equal to 0.05. The protein functional network was performed with STRING 9.0 software (https://string-db.org). Clustering analysis of the DEGs and DAPs was performed using Cluster 3.0 (https://bioservices.capitalbio.com/xzzq/rj/3885.shtml) and the Java Treeview software (https://jtreeview.sourceforge.net). Correlations were analyzed based on the DEGs and  DAPs, and Person correlation tests were conducted for each comparison group, including PM versus PS and PL versus PM.

### Reverse transcription qPCR

The method for total RNA extraction and synthesis of cDNA was described previously. A Bio-Rad CFX96 Touch detection system (Bio-Rad Laboratories, Richmond, CA, USA) with SYBR Green PCR master mix (Aidlab Biotechnologies, Co., Ltd.) was used for qPCR for each sample. In this study, the *EF-1α* gene was used as the internal control gene, which was detected by the de novo transcriptome sequencing of *A. trifoliata* [62]. Primers for qPCR experiments were designed using Primer 5.0 software (Additional file 1: Table S5), and the gene sequences were blasted against the NCBI database. The amplification reactions contained 12.5 μL SYBR Green PCR master mix, 1 μL cDNA, and 0.5 μL of each primer in a final reaction volume of 25 μL. The thermal cycling program began with 3 min at 95 °C, followed by 40 cycles of 95 °C for 10 s and 55 °C for 30 s, with a melt curve from 65 to 95 °C based on increments of 0.5 °C for 5 s. After PCR amplification, quantitative variations were analyzed using the Delta Ct method, and the analysis of statistically significant differences in gene expression was performed by independent samples t-test analysis at *P* < 0.05 using GraphPad Prism 8 software. Correlation analysis between cell wall-related gene and protein expression was performed by Pearson’s correlation coefficient analysis [63].

### PRM analysis

To verify the protein expression levels obtained by TMT analysis, the expression levels of selected proteins were further quantified by PRM analysis. The AQUA stable isotope peptide was spiked in each sample as an internal standard reference. The tryptic peptides were directly loaded on C18 STAGE-tips for desalting prior to reverse-phase chromatography on an Easy nLC-1200 system (Thermo Scientific, USA). Acetonitrile was used for 45 min, increasing from 5 to 35% for a 1-h liquid chromatography gradient, and PRM analysis was performed on a Q Exactive Plus mass spectrometer (Thermo Scientific). The mass spectrometer was operated in positive ion mode, and the full MS1 scan was acquired with a resolution of 60,000 (at 200 m/z). AGC target values and maximum ion injection times were set at 3e6 and 200 ms, respectively; full MS scans were followed by 20 PRMs (MS2 scans) at 30,000 resolution (at m/z 200) with AGC of 3e6 and the maximum injection time set as 120 ms. The targeted peptides were isolated with a 2-Th window. Ion activation/dissociation was performed at a normalized collision energy of 27 in the HCD collision cell. Skyline version 3.7.0 was used to analyze the MS data where signal intensities for individual peptide sequences for each of the significantly altered proteins were quantified relative to each sample and normalized to a standard reference [64].

## Availability of data and materials

The raw sequence data have been deposited in the National Center for Biotechnology Information (NCBI) database (https://www.ncbi.nlm.nih.gov/sra/SRP246017). The mass spectrometry proteomics data have been deposited to the PRIDE partner repository with the dataset identifier PXD017282.

## Supplementary information

**Additional file 1: Fig. S1.** The size distribution of the assembled transcript and unigene sequences of *A. trifoliate.* **Fig. S2.** Volcano plot depicting the transcriptome and proteome data of *A. trifoliate*. a, c Volcano plot depicting the transcriptome and proteome  data in PM_PS. b, d Volcano plot depicting the transcriptome and proteome  data in PL_PM. Absolute log10 and log2 fold changes are plotted on the y-axis and x-axis, respectively. Horizontal dotted line presents *p* values of 0.05 cut-off position while the vertical dotted lines discriminate between genes and proteins having absolute log2 fold change of 1. Red dots represent significantly different upregulated genes. Green dots represent significantly different downregulated genes. Pink dots represent a log2 fold change >1 with *p* < 0.05 in protein expression. Black dots indicate no difference in gene and protein expression. **Fig. S3.** Molecular weight and peptide count distribution were identified from TMT proteomics by searching against the database. a Distribution of the proteins that were identified among different molecular weights. b Distribution of peptide count of the proteins were identified from TMT data. **Fig. S4.** GO and KEGG pathway functional enrichment analysis of co-regulated genes and proteins in *A. trifoliate*. a, b GO enrichment analysis of co-regulated genes and proteins in PM_PS and PL_PM, respectively. c, d KEGG pathways enrichment analysis of co-regulated genes and proteins in PM_PS and PL_PM, respectively. **Table S1.** Sequencing statistics for *A. trifoliate.* **Table S2.** Statistical data of unigenes annotation. **Table S3.** The summary of the total number of transcripts and proteins identified from different stages and replicates. **Table S4.** The summary of the DEGs and DAPs identified from different stages and replicates. **Table S5.** Sequences of specific primers used for qPCR experiment.

## Abbreviations

*Akebia trifoliata*: *A. trifoliata*
TMT: Tandem mass tag
PL: Pectate lyase
PE: Pectinesterase
β-GAL: β-galactosidase
PGs: Polygalacturonase
EXP: Expansin
PS: The non-cracking stage
PM: The initial cracking stage
PL: The total cracking stage
BLAST: Basic Local Alignment Search Tool
NR: Nonredundant protein sequence database
Pfam: Protein families Fdatabase
GO: Gene Ontology database
KEGG: Kyoto Encyclopedia of Genes and Genomes pathway database
DEG: Differently expressed gene
qPCR: Real-time quantitative polymerase chain reaction
PRX: Peroxidase
CAD: Cinnamyl-alcohol dehydrogenase
4CL: 4-coumarate-COA-ligase
HCT: Shikimate *O*-hydroxycinnamoyl transferase
PL: Pectate lyase
PE: Pectinesterase
PG: Polygalacturonase
F26G: Furostanol glycoside 26-*O*-beta-glucosidase
β-GAL: Beta-galactosidase
DAP: Differentially abundant protein
BP: Biological process
MF: Molecular function
CC: Cell component
